# High life satisfaction reported among small-scale societies with low incomes

**DOI:** 10.1073/pnas.2311703121

**Published:** 2024-02-05

**Authors:** Eric D. Galbraith, Christopher Barrington-Leigh, Sara Miñarro, Santiago Álvarez-Fernández, Emmanuel M. N. A. N. Attoh, Petra Benyei, Laura Calvet-Mir, Rosario Carmona, Rumbidzayi Chakauya, Zhuo Chen, Fasco Chengula, Álvaro Fernández-Llamazares, David García-del-Amo, Marcos Glauser, Tomas Huanca, Andrea E. Izquierdo, André B. Junqueira, Marisa Lanker, Xiaoyue Li, Juliette Mariel, Mohamed D. Miara, Vincent Porcher, Anna Porcuna-Ferrer, Anna Schlingmann, Reinmar Seidler, Uttam Babu Shrestha, Priyatma Singh, Miquel Torrents-Ticó, Tungalag Ulambayar, Rihan Wu, Victoria Reyes-García

**Affiliations:** ^a^Institut de Ciència i Tecnologia Ambientals, Universitat Autònoma de Barcelona (ICTA-UAB), Cerdanyola del Vallès, Barcelona 08193, Spain; ^b^ICREA, Barcelona 08010, Spain; ^c^Department of Earth and Planetary Science, McGill University, Montréal, QC H3A0E8, Canada; ^d^Department of Equity, Ethics, and Policy, School of Population and Global Health, McGill University, Montréal, QC H3A 1G1, Canada; ^e^Bieler School of Environment, McGill University, Montréal, QC H3A 2A7, Canada; ^f^Water Systems and Global Change Group, Wageningen University, Wageningen 6700 HB, Netherlands; ^g^International Water Management Institute, Colombo 10120, Sri Lanka; ^h^Instituto de Economía, Geografía y Demografía, Consejo Superior de Investigaciones Científicas, Madrid 28037, Spain; ^i^Institut Metròpoli, Universitat Autònoma de Barcelona, Barcelona 08193, Spain; ^j^Center for Integrated Disaster Risk Management, Pontificia Universidad Católica de Chile, Santiago 8331150, Chile; ^k^College of Agriculture and Environmental Science, University of South Africa, Florida, 1710 Johannesburg, South Africa; ^l^Faculty of Social Sciences, University of Helsinki, Helsinki FI-00014, Finland; ^m^Institute of Resource Assessment, University of Dar es Salaam, Dar es Salaam 16103, Tanzania; ^n^Helsinki Institute of Sustainability Science, University of Helsinki, Helsinki FI-00014, Finland; ^o^Iniciativa Amotocodie, Asunción 1137, Paraguay; ^p^Boliviano de Investigación y de Desarrollo Socio Integral, San Borja, Bolivia; ^q^Instituto Multidisciplinario de Biología Vegetal, Consejo Nacional de Investigaciones Científicas y Técnicas-Universidad Nacional de Córdoba, Córdoba 5000, Argentina; ^r^The Nelson Institute for Environmental Studies, University of Wisconsin-Madison, Madison, WI 53706; ^s^Centre de coopération Internationale en Recherche Agronomique pour le Développement (CIRAD), Unité Mixte de Recherche Savoirs-Environnement-Sociétés (UMR SENS), Montpellier 34398, France; ^t^Department of Nature and Life Sciences, Ibn Khaldoun University, Tiaret 14000, Algeria; ^u^Laboratory of Agro-Biotechnology and Nutrition Research in Semi-Arid Areas, Ibn Khaldoun University, Tiaret 14000, Algeria; ^v^Department of Biology, University of Massachusetts, Boston, MA 02215; ^w^Global Institute for Interdisciplinary Studies, Kathmandu 44600, Nepal; ^x^School of Science and Technology, University of Fiji, Saweni, Lautoka, Fiji; ^y^Global Change and Conservation, Organismal and Evolutionary Biology Research Programme, University of Helsinki, Helsinki FI-00014, Finland; ^z^Zoological Society of London, Mongolia Representative Office, Ulaanbaatar 14201, Mongolia; ^aa^Department of Sociology and Anthropology, Peking University, Beijing 100871, China; ^bb^Norwegian Institute for Cultural Heritage Research, Oslo 0155, Norway

**Keywords:** subjective well-being, happiness, Indigenous Peoples, monetary income, wealth

## Abstract

Global polls have shown that people in high-income countries generally report being more satisfied with their lives than people in low-income countries. The persistence of this correlation, and its similarity to correlations between income and life satisfaction within countries, could lead to the impression that high levels of life satisfaction can only be achieved in wealthy societies. However, global polls have typically overlooked small-scale, nonindustrialized societies, which can provide an alternative test of the consistency of this relationship. Here, we present results from a survey of 2,966 members of Indigenous Peoples and local communities among 19 globally distributed sites. We find that high average levels of life satisfaction, comparable to those of wealthy countries, are reported for numerous populations that have very low monetary incomes. Our results are consistent with the notion that human societies can support very satisfying lives for their members without necessarily requiring high degrees of monetary wealth.

## Significance

It is often said that money can’t buy happiness, yet many surveys have shown that richer people tend to report being more satisfied with their lives. This tendency could be taken to indicate that high material wealth—as measured in monetary terms—is a necessary ingredient for happiness. Here, we show survey results from people living in small-scale societies outside the globalized mainstream, many of whom identify as Indigenous. Despite having little monetary income, the respondents frequently report being very satisfied with their lives, and some communities report satisfaction scores similar to the wealthiest countries. These results imply greater flexibility in the means to achieve happiness than are apparent from surveys that examine only industrialized societies.

One of the most robust findings of happiness research has been a strong correlation between reported life evaluations and income. Life evaluations are commonly measured by asking people for a numerical response to a single question, either the Cantril Ladder, which asks participants to associate their life with a step on an imaginary ladder, or a more straightforward satisfaction with life (SWL) question (1). Life evaluations are frequently taken as holistic measures of well-being and are playing an increasingly prominent role in setting policy agendas (2).

The correlation between income and life evaluation responses has been repeatedly shown for individuals within countries (3–5), as well as at the international scale using Gross Domestic Product (GDP) per capita as a proxy for income (6–9). For example, in the 2022 edition of the World Happiness Report (10), no country with GDP per capita under US$ 4,500 per year reported an average Cantril ladder response above 5.5 (on a scale of 0 to 10). Conversely, average responses exceeding 7 out of 10 were only reported in countries where GDP per capita exceeded US$ 40,000 per year. When statistical models are used to predict the variation among national averages, GDP per capita typically emerges as a dominant predictor of life evaluations (10).

Yet, although life evaluations are frequently correlated with GDP per capita, there are both empirical and theoretical reasons to question the degree to which this reflects a universal predictive relationship. One line of questioning arose from the finding that life evaluations within a country do not consistently increase over time as GDP per capita increases, a phenomenon known as the Easterlin paradox (11, 12). This paradox is often attributed to a combination of adaptation, by which people become accustomed to material wealth over time (13–15), and social comparison, by which people gain satisfaction from their wealth when it appears large relative to others, irrespective of what it represents in absolute terms (16). A complementary line of questioning focuses on the fact that the psychological basis of emotions evolved in ancient times when material wealth was very limited, implying that there should be no direct link to absolute levels of material affluence beyond those required to fulfill basic necessities (17, 18). Happiness research has frequently focused on industrialized societies that are neither historically nor cross-culturally representative (19), potentially biasing the perspective on the relationship between material wealth and life evaluations (20–22). Intriguingly, prior research in a handful of nonindustrialized societies with low levels of monetary wealth and income has shown reports of remarkably high levels of subjective well-being (22–28). Together, these lines of argument challenge the idea that low monetary wealth at the community level should reliably predict low life evaluations, i.e., that material affluence is a requirement for achieving high life evaluations.

Despite these challenges, a recent review states that “the consensus today is that the wealth of nations is closely associated with whether residents can live their lives close to their ideal.” (29). The tacit message implied by this consensus is important for life on Earth. If a high level of material wealth is inherently required for people to live life close to their ideals, achieving high life satisfaction for all humans would presumably require much greater rates of material extraction than at present (30). Policies based on this paradigm are likely to intensify environmental pressures by sacrificing ecosystems to increase material production (31) and are likely to surpass planetary boundaries (32). Given its practical implications, assessing the universality of the wealth-satisfaction link is urgent and requires sampling a wide realm of societies that encompass very different modes of life. Small-scale societies living in close contact with nature, on the fringes of globalized mainstream society, offer distinctly valuable perspectives in this regard.

**Fig. 1.**
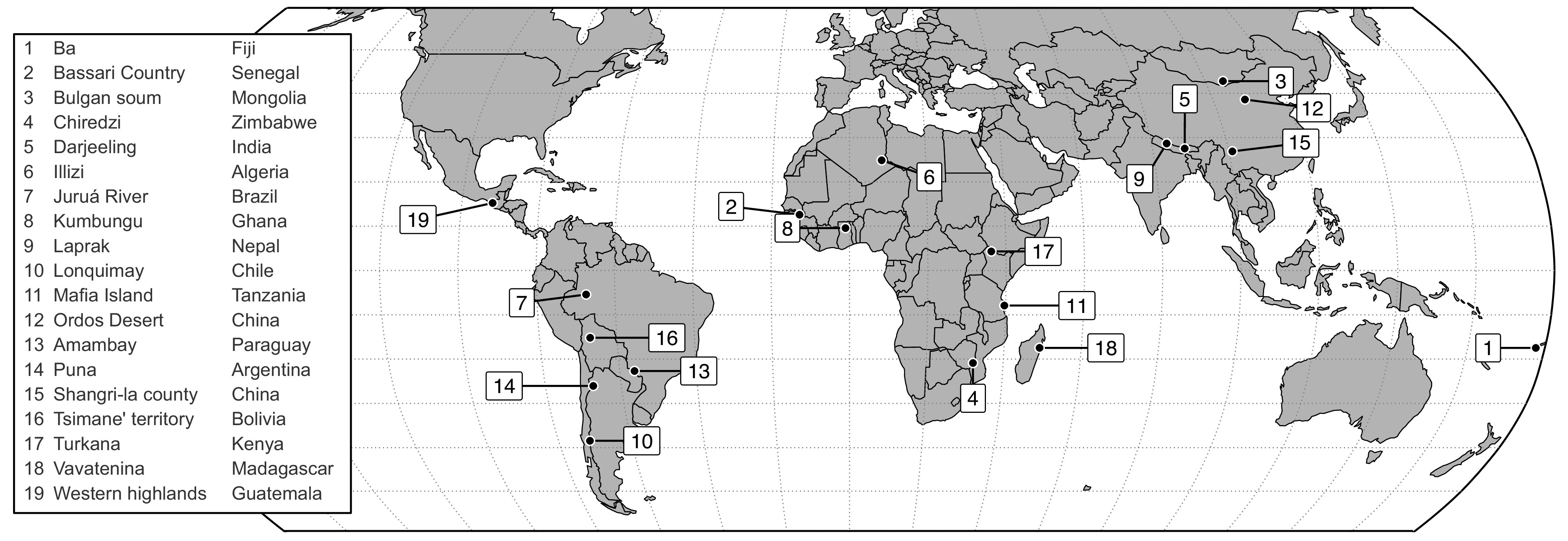
Locations of the study sites. For further details about the study sites, see *Methods*.

Here, we show results from a large, globally coordinated survey including a diverse collection of Indigenous Peoples and local communities at 19 sites spanning five continents (Fig. 1). These small-scale societies were selected for a study designed to assess local knowledge of climate change impacts, and a life evaluation question was included in the standardized survey. All study sites share a strong dependence on nature for livelihoods, but otherwise span a wide range of societal, cultural, and environmental features (33). Following standard practice, we asked survey participants to rate their life satisfaction on a scale of 0 to 10 (*Methods*). Because cash income is typically irregular in societies with nature-dependent livelihoods (34) and only 64% of the households surveyed received any cash at all during the study period, we use the market value of persistent commercial assets as a proxy to estimate monetary income per person (*Methods*).

**Fig. 2.**
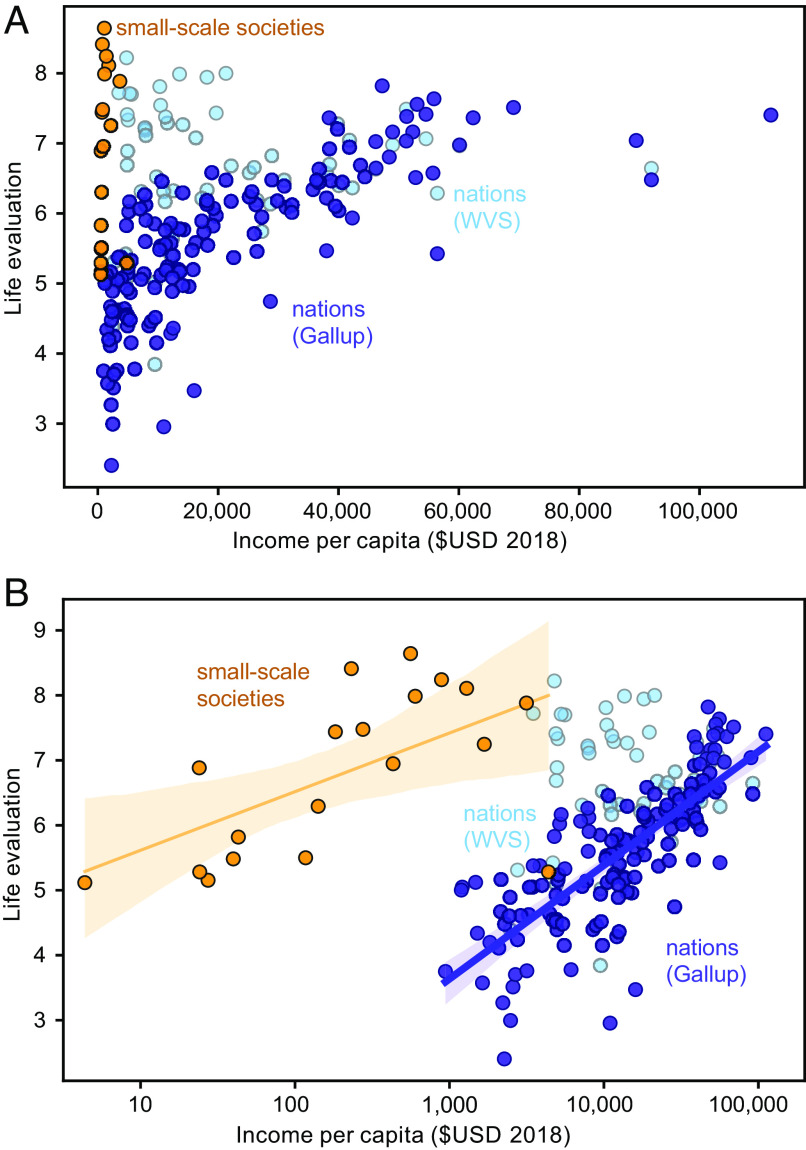
Life evaluations vs. estimated per capita income. Life evaluation scores were reported during personal interviews and averaged over populations. Each dark blue circle shows a national average from the Gallup World Poll (10), with income approximated by GDP per capita. Orange circles show our results from small-scale societies of Indigenous Peoples and local communities with representative incomes estimated from material asset data. Pale blue circles show the rescaled national averages from Wave 7 of the World Values Survey (35). Panel (A) plots the data on linear axes, while (*B*) shows the same data with income on a logarithmic axis and showing OLS linear regressions within 95% uncertainty envelopes. The linear regression for WVS data is not significant (*P* = 0.9) and therefore not shown.

We compare the average reported life satisfaction at each site (*n* = 19 sites; 2,966 participants) with life evaluations obtained by the Gallup World Poll (10), which provides the most comprehensive global coverage of any subjective well-being dataset, including a large number of low-income nations (Fig. 2*A*). The Gallup World Poll results (dark blue circles) show the familiar pattern, whereby high life evaluation responses only occur in high-income countries. In contrast, our small-scale societies report a large range of life satisfaction responses (orange circles), even at low income, and four sites display very high values (>8). The average reported life satisfaction among our 19 surveyed small-scale societies is 6.8 out of 10, even though most of the sites have estimated annual monetary incomes of less than US$1,000 per person. We also include results of the life satisfaction question from the most recent round of the World Values Survey (Wave 7, light blue circles), which includes 40% of the countries sampled by the Gallup World Poll (35). The average life evaluations for Gallup and WVS (World Value Survey) are similar above per capita GDP of USD$25,000 but, notably, the WVS shows a greater proportion of high life evaluations among low-income countries than Gallup.

Caution must be taken with the direct numerical comparison between the life satisfaction and Cantril ladder questions, given that they have been found to produce different answers when asked within the same population. Where they have been directly compared for earlier waves of the WVS and Gallup, the life satisfaction question has been found to systematically result in responses 0.3 to 0.6 points higher than the Cantril Ladder (1, 36–38) with larger discrepancies at low incomes (37). We find that the difference with Gallup is larger for the most recent Wave 7, with an average reported value that is 1.0 higher for the same countries. This discrepancy may reflect a stronger inherent association of income with the Cantril Ladder question, due to the phrasing, which could explain the larger systematic difference at low incomes (37). Regardless, a number of the small-scale societies stand out as reporting particularly high life satisfaction, especially given their very low estimated monetary incomes.

The data shown in Fig. 2*A* are replotted in Fig. 2*B*, with income on a logarithmic axis. The purple regression line shows the highly significant correlation with log(income) that has been previously identified for the Gallup data (*P* < 10^−30^). Although the small-scale societies report much higher life evaluations for a given income than the Gallup data, they are nonetheless significantly correlated with log(income) as well (*P* = 0.01). The WVS Wave 7 data are not significantly correlated with log(income). Intriguingly, the slope of the Gallup regression (1.2, [1.1, 1.4]) overlaps with that of the small-scale societies (0.9, [0.3, 1.5]) within their 95% CIs. But despite this apparent similarity of slopes, the intercepts of the regressions are very different, with the Gallup intercept of 0.2 [0.5, 0.9] falling far below the small-scale society intercept of 4.7 [3.0, 6.2]. In other words, the baseline reported satisfaction among the small-scale societies is much higher, at a given level of income, than implied by the Gallup data.

We conducted further regression analyses of life satisfaction vs. log(income) among the small-scale societies, both within and across villages, in order to test the robustness of the apparent correlation (*Methods*). The results, as summarized in Table 1, reveal statistically significant positive correlations between household income and reported life satisfaction, both at the village level (model 1) and for individual respondents (models 2 to 4). We were not able to identify a strong positive or negative effect of village wealth that would be additive with the individual-level effect (see *SI Appendix*, *Supplementary Table*, Models 6 and 9), although the CIs are relatively wide. Thus, we cannot rule out either a comparison effect (a negative correlation with village wealth), a collective benefit from community-level monetary wealth (a positive correlation with village wealth), or both. Importantly, the individual-level models that do not include village controls (models 2 and 4) account for only a very small fraction of the total variance in individual life satisfaction (*R^2^* = 0.05). In contrast, the model with village control variables explains a much larger fraction of the individual variance (*R^2^_adj_* = 0.35), indicating that village-level characteristics which are unrelated to average monetary wealth are responsible for a greater proportion of the variations in individual life satisfaction than the estimated individual wealth. In fact, when these village-level characteristics are included, individual monetary wealth does not provide significant additional predictive power for reported life satisfaction (*SI Appendix*, *Supplementary Table*, Models 5 and 8).

**Table 1.** Regression models of life satisfaction among small-scale societies

	Villages		Respondents	
Model	1	2	3	4
Village average log(assets/capita)	0.25* (±0.09)			−0.07 (±0.1)
Respondent household log(assets/capita)		0.19* (±0.07)	0.11* (±0.03)	**0.24**^†^ (±0.04)
Constant	5.6 (±0.7)	5.7 (±0.3)	6.4 (±0.2)	5.9 (±0.6)
Village control included			yes	
R^2^(adj)	0.1	0.05	0.35	0.05

The first two rows give the regression coefficients for the predictor variables specified at the left hand side. Model 1 explains village average life satisfaction, whereas models 2-4 explain individual-level life satisfaction. Coefficients are in boldface where statistically significant (*P* < 0.1) with *and ^†^indicating high significance (*P* < 0.01) and very high significance (*P* < 0.001), respectively. Values in parentheses indicate 1 SE ranges.

Taken together, our results suggest that greater material affluence can tend to be associated with more positive life evaluations within the context of small-scale societies, as has been widely identified in industrialized societies. However, the variations in individual wealth explain only a very small part of the variation in life satisfaction, and the most robust aspect of our findings is that most of the surveyed small-scale societies achieve much higher levels of satisfaction, at a given level of wealth, than the Gallup national averages.

**Fig. 3.**
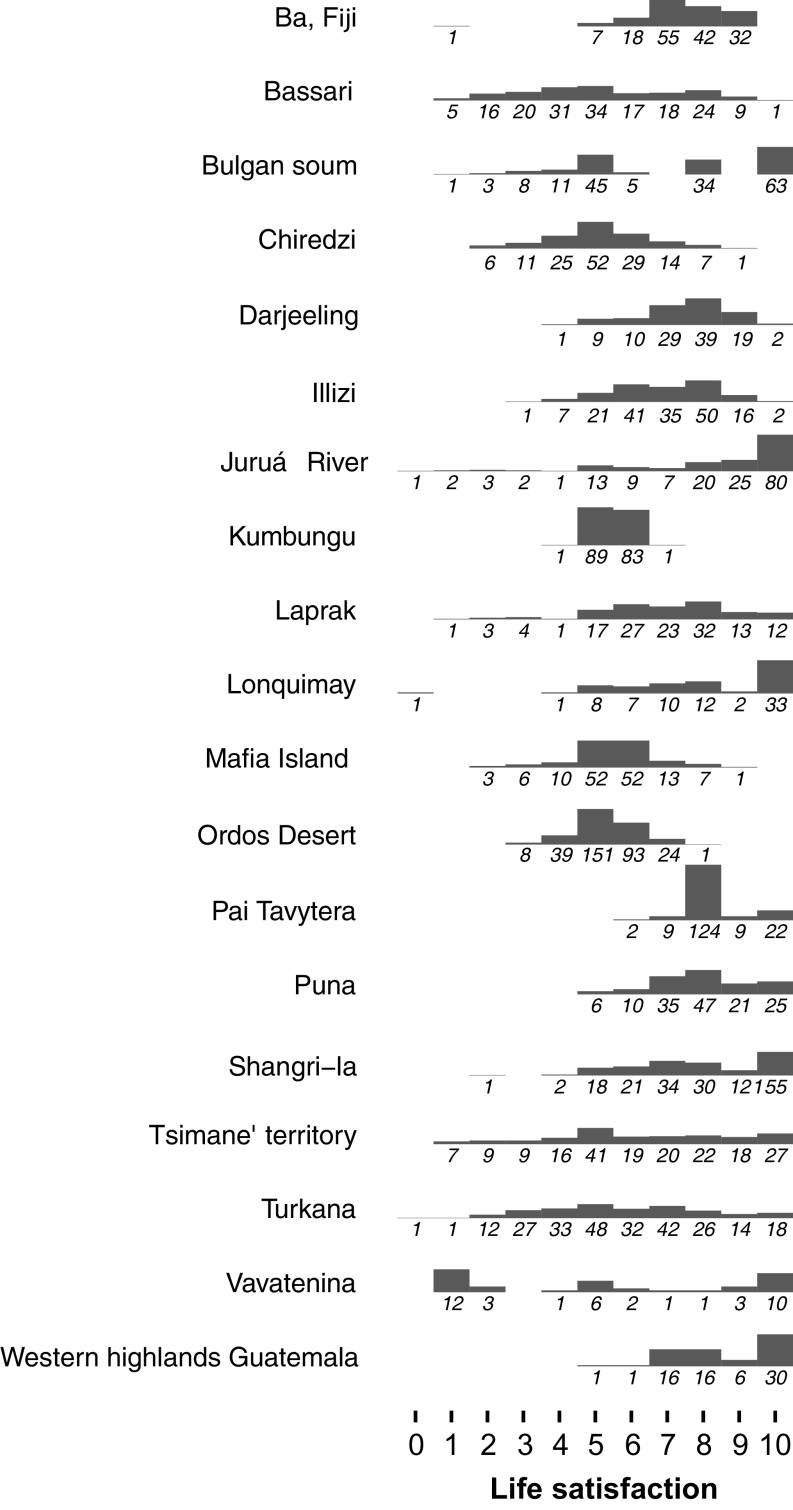
Responses to the life satisfaction question, by site. The number of responses for each numerical score is shown in italics and represented visually as histograms.

The answers provided to life satisfaction questions have been shown to depend on numerous factors that vary by society (38–41). Of potential concern to our work is the fact that, at some of the study sites, respondents might make relatively infrequent use of numerical scales in their daily lives. However, subsets of the study communities that used visual aids for the numerical scale, or where leading examples were provided, did not differ significantly in average life satisfaction (*Methods*). In addition, prior work among minimally monetized communities found consistent relationships between reported positive affective states and life satisfaction (26). Furthermore, although ref. 42 has reported response patterns which emphasize top, middle, and bottom values on the scale in certain population segments (the “focal values” problem), no systematic pattern is apparent across the distributions we collected (Fig. 3). Given the absence of obvious methodological inconsistency, we do not see any reason to consider the responses obtained in our survey to be less reliable than the responses provided to other international polls.

Our results show that remarkably high measures of subjective well-being are widespread among the 19 small-scale societies studied. Qualitatively similar results have been previously found by independent studies in Australia (43), Tanzania (24), Alaska (28) and the western tropical Pacific (26). It is important to note that life satisfaction is not uniformly high at all of our sites, with some sites reporting averages as low as 5.1. This might be expected, given that some of these communities face hardships in meeting basic needs and ensuring health (44), and many have endured oppression, disenfranchisement, and marginalization (45). These pressures can exacerbate legacies of intergenerational trauma and reduced cultural engagement, leading to declines in peoples’ mental well-being (46). Nonetheless, high life satisfaction appears to be commonly reported among the small-scale societies that have been surveyed, despite very low rates of monetary income.

Prior work has empirically linked life evaluation to factors that have no inherent market cost and could therefore support high life satisfaction independently of monetary wealth or income. For example, social support, trust, and freedom are widely identified as important factors, as is the absence of corruption (38, 47, 48). Nonmaterial factors such as these have been specifically identified among Indigenous Peoples and local communities. For contemporary Inuit of the Arctic, happiness has been associated with family relations and social participation (49), while relations with other people, with nature, and with spiritual beings appear central for the subjective well-being of other Native Americans (50), Amazonian Indigenous communities (51) and Aboriginal Australians (52). Among numerous Andean Indigenous Peoples, concepts of “the good life” or “living well” emphasize the importance of maintaining harmonious interpersonal relationships, some of which extend to nonhumans and spirits (53, 54). Observational studies in western societies have also suggested that spending time in natural surroundings raises life satisfaction (55), which may play an additional role at our sites.

The striking aspect of our findings, particularly compared to the widely cited Gallup World Poll, is that reported life satisfaction in very low-income communities can meet and even exceed that reported at the highest average levels of material wealth provided by industrial ways of life. This is at odds with the consensus view given in ref. 29, but is consistent with previous cross-cultural studies of subjective well-being suggesting that most people are fairly happy by default (23, 56). It also underscores the dominant role that nonmaterial factors, such as social support and trust, could play in raising the future happiness of peoples across the world (57).

Our findings provide strong empirical support for the argument that achieving high reported life satisfaction does not require the elevated rates of material consumption generally associated with high monetary income. Instead, they add weight to the importance of identifying the underlying factors that cause people to report high satisfaction with their lives. It has long been known that nonmonetary factors are important to well-being—the insight here is that those factors can yield higher levels of satisfaction, at the population level, than typically thought. Further research into the factors supporting high levels of life satisfaction while maintaining low material requirements, as exemplified by the communities studied here, may provide unexplored strategies to improve the well-being of humans while navigating planetary boundaries.

## Methods

The data presented here were collected through a total of 2,966 in-person interviews, carried out among 19 globally distributed sites in 18 countries. All surveys were part of the Local Indicators of Climate Change Impacts (LICCI) project, which aims to bring insights from Indigenous and local knowledge systems to climate research and followed a standardized protocol for data collection (33).

### Sample Selection.

All information reported pertains to Indigenous Peoples and local communities, defined according to the Intergovernmental Science-Policy Platform on Biodiversity and Ecosystem Services. These communities exist in rural areas in close interaction with the environment and are supported largely through use of nature.

The sites included here represent a subset of those included in the LICCI project, which were chosen according to their suitability to contribute to the LICCI goals of gathering local knowledge on climate change and impacts (33). Site selection was guided by the following criteria: prioritizing locations with deficient instrumental data and limited studies on local knowledge of climate change impacts; considering climate types to include representatives from the five main Köppen-Geiger-defined categories (tropical, arid, temperate, continental, and polar/cold); focusing on four nature-based livelihood activities (agriculture, fishing, pastoralism, and foraging); and assessing feasibility based on logistical considerations for establishing an extended network of partners responsible for data collection (33). These criteria resulted in a geographically dispersed and highly diverse collection of sites. To engage local partners, a widely circulated call emphasized criteria such as research experience and previous relations with the proposed study site, with a particular encouragement for South-based researchers to apply.

In each designated site, one research partner undertook the responsibility of gathering data across 3 to 5 villages, the lowest administrative unit in an area normally overseen by a village leader. The chosen villages were intended to be representative and relatively uniform in terms of both the environmental and sociocultural conditions specific to the site (33). Villages exhibiting atypical circumstances, such as those with significant donor intervention, were deliberately excluded. Additionally, for logistical efficiency, only villages with more than 20 households were selected for sampling, while those with more than 500 households were subdivided into smaller units.

To choose households, partners employed a simple random sampling, selecting households from a local census (33). The survey incorporated questions directed at both the household as a whole and the individuals within it (see *SI Appendix* for the survey questions). Household-related inquiries were addressed to anyone recognized as a household head, defined as the individual with the authority—whether through formal or informal means—to make decisions regarding the allocation of household labor and resources. Within each household, partners relied on convenience quota sampling among household heads to select one or two participants to independently answer individual questions, aiming for an approximately equal overall representation across gender and age categories.

### Survey.

Interviews were carried out in person, in the local language, by researchers working together with local assistants. The Gallup World Poll uses a similar in-person interview strategy for low-income countries where many people cannot read or write. All partners were trained to use the same protocol during three 1-week in-person workshops held in 2019. At all sites local partners worked with interpreters who helped test the surveys, identifying possible points of confusion, and selecting the best wording. All questions were then tested with 10 people and adjusted if needed. The surveys included many sections, within which the life satisfaction question was consistently placed following questions about the household demographic and economic state, the local environment, dependence on natural resources, and the challenges of climate change. The length of interviews ranged between 40 and 90 min.

The SWL question was translated from “Considering all aspects, how satisfied are you with your life on a scale from 0 to 10?”. Variants of this question have been widely used for decades, in many languages, including the German Socio-Economic Panel, the British Household Panel Survey, and the World Values Survey (see ref. 1 for an overview). The SWL question is shorter than the Cantril Ladder question, which is also frequently referred to as measuring life satisfaction, though the two questions are more accurately described as measuring life evaluation. Both questions have been validated across many cultures (38, 58).

Table 2 provides details on variations in how the life satisfaction question was adjusted to ensure comprehension among the different sites. At some sites, the question was immediately understood and no further expansion was given. For a subset of sites, additional expansion was provided in order to better clarify the intent of the question, for example anchoring the ends of the 11-point scale by contrasting “a very fine, happy life” with an “unsatisfying, heavy and difficult life.” For a small subset of sites, specific examples were given of life experiences that might be considered satisfying or unsatisfying (e.g., good/bad health, family problems, etc.). In addition, given that the abstract numerical scale appeared unintuitive for some of the participants at some sites, the interviewers sometimes chose to use a visual aid for some or all of the participants. In this case they employed a line, which they described as showing a range from least satisfied at one end to most satisfied at the other end, on which the participant was asked to indicate their personal life satisfaction. Researchers then assigned a score based on the position on the line indicated by the participant.

**Table 2.** Sites included in the study

Label	Site	Country	Group Name	Verbal expansion	Visual aid	Examples	n	SWL mean	SWL std	Household assets USD mean	Household assets USD std	Per capita assets USD mean	Per capita assets USD std
1	Ba	Fiji	iTaukei	No	No	No	155	7.4	1.2	$1655	$6366	$183	$669
2	Bassari Country	Senegal	Bassari	Yes	Yes	No	175	5.2	2.2	$484	$699	$27	$34
3	Bulgan soum	Mongolia	Mongolian	Yes	No	No	170	7.2	2.6	$10988	$10354	$1680	$1905
4	Chiredzi	Zimbabwe	Farmers	Yes	No	Yes	145	5.1	1.4	$71	$36	$4	$2
5	Darjeeling	India	Singalila	Yes	No	Yes	109	7.5	1.2	$673	$1365	$275	$876
6	Illizi	Algeria	Tuareg	Yes	No	No	173	6.9	1.4	$353	$602	$24	$30
7	Juruá River	Brazil	Riberinhos	Yes	Yes	No	163	8.4	2.2	$2213	$998	$232	$120
8	Kumbungu	Ghana	Dagomba	No	No	No	174	5.5	0.5	$1299	$1923	$40	$45
9	Laprak	Nepal	Gurung	Yes	No	No	133	6.9	1.9	$3311	$2340	$431	$331
10	Lonquimay	Chile	Mapuche-Pehuenche	Yes	Yes	No	74	8.1	2.1	$7853	$4413	$1291	$1011
11	Mafia Island	Tanzania	Fisherfolk	Yes	Yes	Yes	144	5.5	1.2	$1358	$1586	$117	$156
12	Ordos Desert	China	Mongolians	Yes	Yes	Yes	316	5.3	0.9	$32157	$9325	$4376	$2250
13	Amambay	Paraguay	Pai Tavytera/Guarani	No	No	No	166	8.2	0.8	$5237	$4214	$888	$866
14	Puna	Argentina	Kolla-Atacameños	Yes	Yes	No	144	8.0	1.3	$4013	$1858	$600	$269
15	Shangri-la county	China	Tibetan	No	No	No	174	7.9	1.8	$42448	$28882	$3149	$2444
16	Tsimane' territory	Bolivia	Tsimane'	No	No	No	188	6.3	2.5	$1360	$1107	$141	$131
17	Turkana	Kenya	Daasanach	No	No	No	254	5.8	2.2	$471	$792	$43	$83
18	Vavatenina	Madagascar	Betsimisaraka	Yes	No	No	39	5.3	3.7	$199	$267	$24	$30
19	Western highlands	Guatemala	Farmers	No	No	No	70	8.6	1.4	$3503	$5318	$560	$887

n = number of participants at the site, SWL = satisfaction with life, std = standard deviation. See *Materials and Methods* for discussion of the Verbal expansion, Visual aid, and Examples.

Because these methodological variations could conceivably introduce bias into the responses, we compared the means of the subpopulations. We found that the sites at which visual aids were used had a lower mean SWL (6.7, n = 6) than the remaining sites (6.8, n = 13), but the difference was not statistically significant. Similarly, the sites at which the question was expanded upon had a lower mean SWL (6.6, n = 11) than those at which it was only asked verbatim (7.1, n = 8), which was not statistically significant. Finally, the sites at which specific examples were provided had a lower mean SWL (5.8, n = 4) than the sites at which examples were not provided (7.1, n = 15) which, again, was not statistically significant. Based on these comparisons, there is no evidence that the high reported SWL values represent an artifact based on how the question was posed.

### Estimation of Monetary Income.

A criterion for LICCI site selection was high dependence on nature for livelihood (33). As a result, income from wage labor and sales is often sporadic among the communities surveyed, and record-keeping of income is rare. Many activities are oriented toward supplying household needs, for which income and consumption can be confounded (34). In the aim of capturing long-term average incomes, we used the monetary value of commercial physical assets of study participants and then converted these to income using a range of asset:income ratios. We focused on commercial physical assets despite the fact that other locally produced assets may have a market price (e.g., livestock, plant-made assets such as canoes or utensils) because they are rarely purchased within these communities.

We calculated a representative monetary value of household commercial assets using the values for new equivalent goods in the local market. To select assets that capture variation in ownership of market assets across households in a site, we drew on participant observation and interviews with knowledgeable people in the village to first select a list of 15 assets that reflected such variation. We identified the 5 assets with the highest market values, owned by a few households (e.g., motorbike, refrigerator, television, tractor), 5 high-value assets owned by a large fraction of the community (e.g., shotgun, fishing net, mobile phone) and 5 of the most common assets (e.g., machete, cooking pot). We tested variation in assets ownership by including the 15 assets in a draft version of the survey that was tested with 10 households. We then selected the 10 assets displaying most variation across households in each site. We also collected the prices of these assets in the local market. Household wealth was calculated as the product of the number of assets owned by the household, multiplied by the local market price of a new asset (33).

**Fig. 4.**
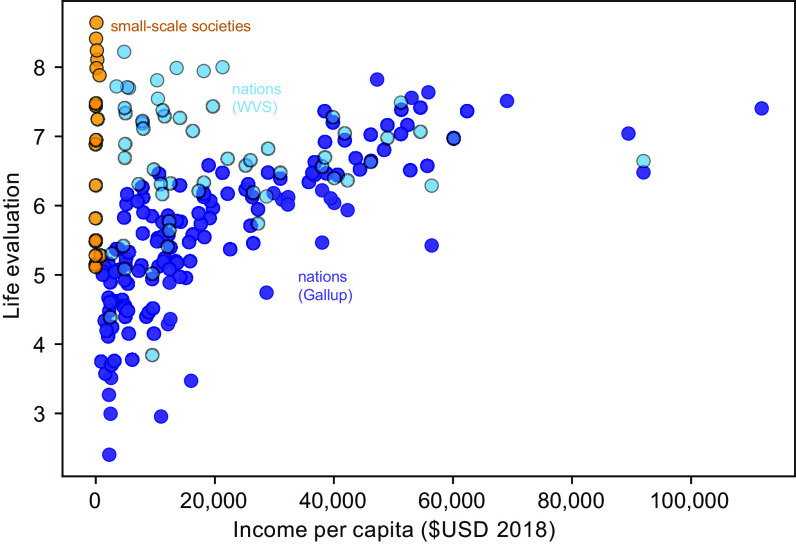
Life satisfaction vs. estimated income using a higher asset:income ratio. As in Fig. 2*A*, but assuming a value of 5 for the asset:income ratio among our study sites, more similar to asset:income ratios estimated for industrialized countries (49).

For comparison with national incomes estimated from GDP per capita, we converted the individual asset values to estimated annual income using a fixed ratio for all communities and divided by household size. Fig. 4 assumes an asset:income ratio of 1. This value is small compared to typical values for asset:income, which have been found to vary from ~5 to ~7 at the national level (59), but was chosen as a very conservative value given the fact that people in the studied communities tend to primarily purchase goods of low durability (e.g., food, clothing). Fig. 4 shows the equivalent of Fig. 2*A* using an asset:income ratio of 5, an assumption under which the occurrence of high life satisfaction at low levels of wealth is far more pronounced.

The magnitude of incomes estimated here is similar to income estimates made by the Poverty Environmental Network (PEN) study. The PEN study estimated income for 33 tropical communities that bore many similarities with the communities studied here (34). Across these communities, the average annual cash income from wages and business was $505 USD per adult (purchasing power parity, PPP). Accounting for inflation, this is equivalent to $593 USD per adult in 2018 (PPP). For comparison, the average for all sites in our dataset is $1559 USD per person (PPP, 2018) using an asset:income ratio of 1, or $520 USD per person using an asset:income ratio of 3.

**Fig. 5.**
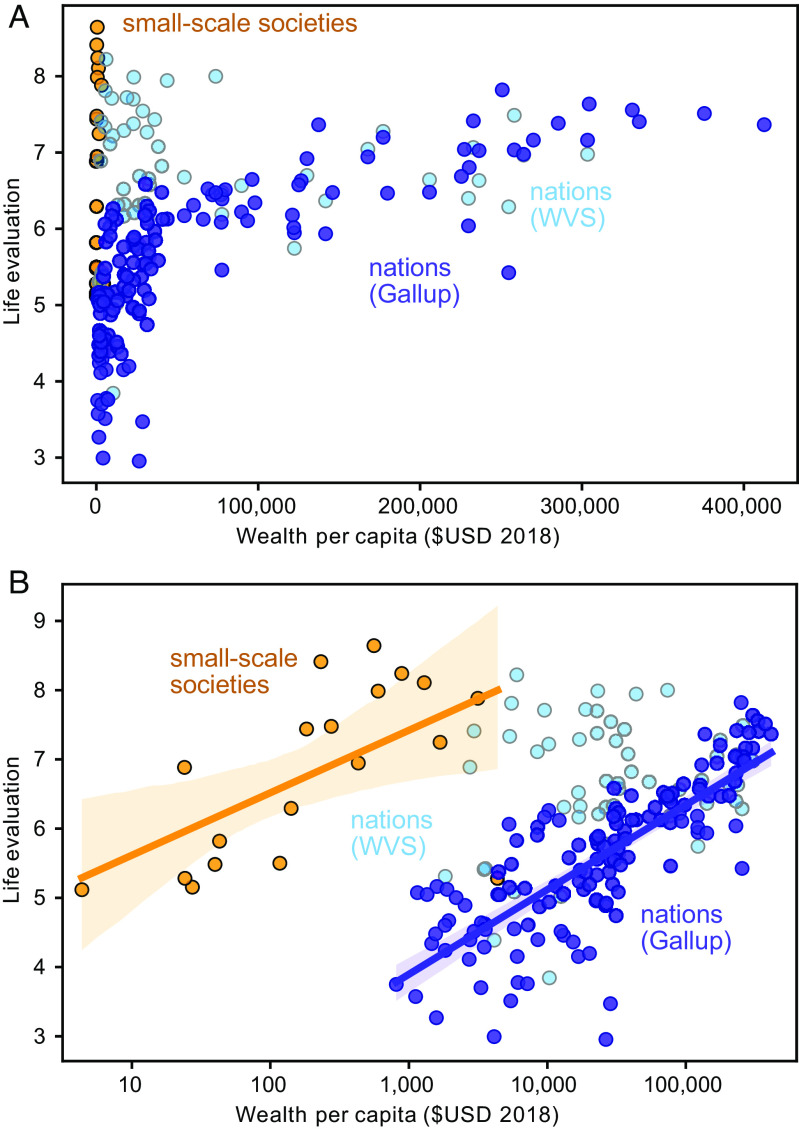
Life satisfaction vs. estimated wealth. As in Fig. 2, but for estimates of wealth, rather than income. Dark blue symbols show national averages for the Gallup World Poll and pale blue symbols show national average for the WVS wave 7, both using the produced capital measure from ref. 60. Orange symbols show our results for small-scale societies. Panel (A) plots the data on linear axes while panel (*B*) shows the same data with wealth on a logarithmic axis.

Ref. 34 also estimated the values of nonmonetary income sources. When including imputed values of crops, livestock and other income, the average income was $1326 USD per adult, and when estimated values for forest and other environmental products were also included the average total income was $1852 USD per adult. Accounting for inflation, this is equivalent to $2174 USD per adult in 2018 (PPP). An equivalent comparison to that shown in Fig. 2 (life satisfaction vs. income) is given for wealth in Fig. 5, showing that the overall comparison is very robust regardless of whether the estimate of wealth or income is used.

### Comparison with Gallup World Poll and WVS Results.

Gallup World Poll asks the Cantril Ladder question: “Please imagine a ladder with steps numbered from 0 at the bottom to 10 at the top. The top of the ladder represents the best possible life for you and the bottom of the ladder represents the worst possible life for you. On which step of the ladder would you say you personally feel you stand at this time?” The WVS Wave 7 asks the question: “All things considered, how satisfied are you with your life as a whole these days? Using this card on which 1 means you are ‘completely dissatisfied’ and 10 means you are ‘completely satisfied’ where would you put your satisfaction with your life as a whole?”. In order to compare the 10-point WVS answer with the 11-point scale used in our survey and by Gallup, we linearly rescaled the WVS responses by subtracting 1 and multiplying by 1.11.

### Regression Analysis.

We regressed the life satisfaction scores against estimated household incomes (total and per capita) at both the village and individual levels for the 2,814 respondents for which household income estimates were available. For the individual-level regressions, we tested the effect of village dummy variables as well as the village average income. The estimates in Table 1 were made using Ordinary Least Squares (OLS). Following a common convention in the literature, we also estimated ordered logit models for the respondent-level data. These are presented in *SI Appendix*, Table S1 and show qualitatively identical findings. The ordered logit model drops the assumption that the meanings (i.e., the latent well-being value) of different life satisfaction responses are equally spaced. *SI Appendix*, Table S1 shows raw coefficients, which are quantitatively comparable with OLS coefficients for small values [marginal effects can be calculated more precisely by transforming the raw coefficients *f* to exp(*f*)-1].

The results are significant at the 1% level or higher for all models that use the household income per capita. Regression slopes range from 0.10 to 0.25 and are largely consistent across the two measures of income, as well as the two types of model. The variance explained by individual-level differences is small compared with the village-level variance, as estimated from a model with only village dummy variables. This result suggests that more of the variance in individual life satisfaction is related to characteristics that are common to a village than to individual income and that these characteristics are unrelated to the average income of the village.

### Ethics and Inclusion.

#### Ethics approval.

The research protocol received approval from the Ethics Committee of the Universitat Autonoma de Barcelona (CEEAH 4781), and the LICCI project adheres to the ethical guidelines of the European Research Council. An external and independent ethics advisor thoroughly reviewed all procedures and documents, providing continuous feedback to the team and reporting to the project funders. As outlined in the protocol, prior to commencing data collection, all partners were required to secure Free, Prior and Informed Consent (FPIC) from both the organizations representing the communities and the individual respondents (33). During the initial village visit, partners conducted a meeting to introduce the research and seek consent to stay in the village. In these meetings, detailed information about the study’s objectives and scope, participant involvement, as well as the associated costs and benefits was presented. Written consent was obtained from the community as a whole and individual FPIC was sought from each participant.

#### Inclusion in the research process.

Local researchers were involved throughout the research process. The LICCI research project was carried out through a partnership of a core team of 10 researchers working at ICTA-UAB (host institution) and 45 international partners. To recruit partners, a call was widely circulated, which encouraged South-based researchers to apply. Priority was given to partners with strong preexisting links with the sites where the study was conducted, as well as those planning field work of greater than 12 mo duration. For the 19 sites featured here, 13 partners collected data in their own countries, and two self-identify as Indigenous scholars. Among the six partners whose origin differred from the country in which they collected data, one had worked in the study site for more than 5 y, another for more than 10 y, and four had planned a 1-y field work in the proposed site.

The core team designed the study and drafted the data collection instruments. All partners attended a 1-wk face-to-face training at the host institution, during which data collection instruments were refined and adapted to specific cases (33). Through the training, partners were acquainted with the project’s rationale, received comprehensive explanations regarding the implementation of all data collection protocols, and had the opportunity to engage in discussions about practical aspects. The training encompassed discussions on the ethical considerations surrounding the incorporation of Indigenous knowledge in research and the elaboration of a “Local Knowledge Research Agreement” intended for discussion and negotiation with communities. During the training workshop, partners also had the opportunity to discuss issues related to data ownership and sharing.

Partners implemented the study in their selected site. Ownership of individual datasets rests with the partners who collected the data, who can publish them without the core team. Researchers of the core team leading collective publications invite any partner whose data are being used (a document with publication policies is available upon request).

#### Benefit sharing.

In community meetings, partners facilitated an open dialogue that resulted in the establishment of a “community engagement protocol.” In this dialogue, participants were given the opportunity to express their preferences on how they wished the information to be returned and communicate any additional requirements they may have had. Information has been returned to the site through community meetings, meeting local leaders, seminars at the local host institutions (i.e., local universities or Non-Governmental Organizations hosting the partner), and/or the production of media (film or books).

## Supplementary Material

Appendix 01 (PDF)

## Data Availability

Anonymized survey data have been deposited at ref. 61.
