# Supplementary material for: High life satisfaction reported among small-scale societies with low incomes
Source: Proc Natl Acad Sci U S A. 2024 Feb 5;121(7):e2311703121. doi: 10.1073/pnas.2311703121 (PMC10873637; doi:10.1073/pnas.2311703121)
Supplement: Supplementary file 1 — Appendix 01 (PDF) [file pnas.2311703121.sapp.pdf]

PNAS

Supporting Information for

High life satisfaction reported among small-scale societies with low incomes

Eric D. Galbraith, Christopher Barrington-Leigh, Sara Miñarro, Santiago Álvarez-Fernández, Emmanuel M. N. A. N. Attoh, Petra Benyei, Laura Calvet-Mir, Rosario Carmona, Rumbidzayi Chakauya, Zhuo Chen, Fasco Chengula, Álvaro Fernández-Llamazares, David García-del-Amo, Marcos Glauser, Tomas Huanca, Andrea E. Izquierdo, André B. Junqueira, Marisa Lanker, Xiaoyue Li, Juliette Mariel, Mohamed D. Miara, Vincent Porcher, Anna Porcuna-Ferrer, Anna Schlingmann, Reinmar Seidler, Uttam Babu Shrestha, Priyatma Singh, Miquel Torrents-Ticó , Tungalag Ulambayar, Rihan Wu, and Victoria Reyes-García

Email: victoria.reyes@uab.cat

This PDF file includes:

Table S1

Table S1. Small-scale society regression models of life satisfaction. Each column of the table reflects one model variant, numbered (1) through (15). Models (1) and (2) explain village-level mean life satisfaction, while the remainder explain individual-level life satisfaction and differ according to whether they use village means, individual values and/or dummy variables as predictors. Models shown as having 'village controls' include categorical 'dummy' variables for the 73 villages. Models (1) through (9) are ordinary least squares (i.e. linear models), whereas (10) through (15) are ordered logistic regression models. The first four rows give the regression coefficients for the specified predictors. Shading indicates significance level, values in parentheses give 1 SE.

	Villages		Respondents												
	OLS		OLS							Ordered logit					
Model	(1)	(2)	(3)	(4)	(5)	(6)	(7)	(8)	(9)	(10)	(11)	(12)	(13)	(14)	(15)
**Mean log(HH income)**	***.25***					.056						.038			
	(.095)					(.096)						(.089)			
**Mean log(HH income/capita)**		**.25⋆**							−.073						−.080
		(.085)							(.097)						(.091)
**Log(HH income)**				***.19***	**.14†**	**.14†**				***.14***	**.15†**	**.11†**			
				(.070)	(.034)	(.034)				(.065)	(.034)	(.028)			
**Log(HH income/capita)**							**.19⋆**	**.11⋆**	**.24†**				***.15***	**.10⋆**	**.20†**
							(.066)	(.034)	(.038)				(.062)	(.033)	(.035)
**constant**	**5.1†**	**5.6†**	**7.0†**	**5.3†**	**6.0†**	**5.2†**	**5.7†**	**6.4†**	**5.9†**						
	(.71)	(.45)	(3e-12)	(.48)	(.25)	(.63)	(.29)	(.18)	(.62)						
**/cut1**										**−5.9†**	**−4.4†**	**−5.8†**	**−6.2†**	**−5.1†**	**−6.5†**
										(.75)	(.74)	(.85)	(.64)	(.70)	(.85)
**/cut2**										**−3.5†**	**−1.95†**	**−3.4†**	**−3.8†**	**−2.6†**	**−4.1†**
										(.52)	(.43)	(.65)	(.38)	(.39)	(.67)
**/cut3**										**−2.4†**	***−.85***	**−2.4†**	**−2.7†**	**−1.50†**	**−3.0†**
										(.51)	(.36)	(.65)	(.34)	(.31)	(.66)
**/cut4**										**−1.68†**	−.058	***−1.61***	**−1.95†**	***−.70***	**−2.3†**
										(.50)	(.38)	(.64)	(.33)	(.33)	(.65)
**/cut5**										*−.93+*	***.75***	−.87	**−1.21†**	.11	***−1.52***
										(.48)	(.37)	(.62)	(.30)	(.30)	(.62)
**/cut6**										.33	**2.3†**	.40	.062	**1.65†**	−.25
										(.43)	(.36)	(.57)	(.25)	(.28)	(.57)
**/cut7**										***.98***	**3.3†**	*1.05+*	**.72⋆**	**2.6†**	.41
										(.43)	(.40)	(.57)	(.27)	(.31)	(.56)
**/cut8**										**1.50†**	**4.1†**	**1.57⋆**	**1.24†**	**3.4†**	.93
										(.44)	(.42)	(.58)	(.28)	(.34)	(.57)
**/cut9**										**2.4†**	**5.3†**	**2.5†**	**2.1†**	**4.7†**	**1.83⋆**
										(.46)	(.50)	(.60)	(.30)	(.43)	(.59)
**/cut10**										**2.9†**	**5.9†**	**2.9†**	**2.6†**	**5.3†**	**2.3†**
										(.46)	(.50)	(.60)	(.31)	(.43)	(.60)
**/cut11**										**2.9†**	**5.9†**	**3.0†**	**2.6†**	**5.3†**	**2.3†**
										(.46)	(.50)	(.60)	(.31)	(.43)	(.60)
**Village controls**			Yes		Yes			Yes			Yes			Yes	
R^2	.086	.11	.37	.035	.37	.035	.046	.37	.047						
R^2**(adj)**	.073	.095	.35	.034	.35	.034	.046	.35	.047						
**pseudo-**R^2										.007	.12	.007	.009	.12	.010
**obs.**	73	73	2814	2814	2814	2814	2814	2814	2814	2814	2814	2814	2814	2814	2814
$\mathbf{N}_{\text{clusters}}$			73	73	73	73	73	73	73	73	73	73	73	73	73
**log likelihood**	−129	−128	−5493	−6084	−5482	−6083	−6067	−5487	−6065	−5769	−5100	−5769	−5752	−5105	−5750

Significance: **0.1%†** **1%⋆** ***5%*** *10%+*
